# Associations between serum total cholesterol level and bone mineral density in older adults

**DOI:** 10.18632/aging.204514

**Published:** 2023-02-10

**Authors:** Sheng Hu, Silin Wang, Wenxiong Zhang, Lang Su, Jiayue Ye, Deyuan Zhang, Yang Zhang, Qiang Guo, Dongliang Yu, Jinhua Peng, Jianjun Xu, Yiping Wei

**Affiliations:** 1 Department of Thoracic Surgery, The Second Affiliated Hospital of Nanchang University, Nanchang, China

**Keywords:** total cholesterol, bone mineral density, negative association, multiple equation regression analysis, smooth curve fitting

## Abstract

Background: Osteoporosis is a major clinical problem in elderly men and women. The correlation between total cholesterol and bone mineral density remains controversial. NHANES is the cornerstone for national nutrition monitoring to inform nutrition and health policy.

Methods: Sample sizes and the location of the study and the time when it was conducted: we obtained 4236 non-cancer elderly from NHANES (National Health and Nutrition Examination Survey) database from 1999 to 2006. Data were analyzed with the use of the statistical packages R and EmpowerStats. We analyzed the relationship between total cholesterol and lumbar bone mineral density. We performed research population description, stratified analysis, single factor analysis, multiple equation regression analysis, smooth curve fitting, and threshold effect and saturation effect analysis.

Results: A significant negative association between serum cholesterol levels and bone mineral density of the lumbar spine in US non cancer affected older adults aged 60 years or older. Older adults ≥ 70 years of age had an inflection point at 280 mg / dl, and those with moderate physical activity had an inflection point at 199 mg / dl, The smooth curves they fitted were all U-shaped.

Conclusions: There is a negative association between total cholesterol and lumbar spine bone mineral density in non-cancer elderly greater than or equal to 60 years of age.

## INTRODUCTION

Makovey and Chen [1] studied the relationship of cholesterol and bone mineral density in women before and after menopause with a modest inverse correlation. And Yang, Liu [2] found an inverse correlation between bone mineral density and serum cholesterol levels in type 2 diabetes. Ackert-Bicknell [3] found an inverse correlation between serum HDL levels and bone status in vitamin D-deficient postmenopausal women. However, these are small population-specific studies. A study by Tang et al. [4] observed a negative correlation between high-density lipoprotein cholesterol (HDL-C) levels and BMD. In contrast, Zolfaroli et al. [5] concluded that HDL-C levels were positively correlated with BMD in the lumbar spine and femoral neck in a postmenopausal female population. In addition to this, Cui et al. [6] concluded that HDL-C levels in premenopausal and postmenopausal subjects were not associated with BMD at any site. The conclusions of these studies remain controversial. And, what is the relationship between serum total cholesterol and bone mineral density in normal people without diabetes and cancer? Among the normal elderly without diabetes and cancer? In the large sample of normal elderly without cancer? In a large sample of normal older adults without cardiovascular disease? Hence, the relationship between cholesterol levels and BMD, and whether cholesterol levels have potential value in predicting osteoporosis is worth exploring.

Therefore, our aim in this study was to evaluate the relationship between total cholesterol and bone mineral density using a representative sample of normal elderly without diseases such as cancer in the national health and Nutrition Examination Survey [7] (NHANES). NHANES is the cornerstone for national nutrition monitoring to inform nutrition and health policy. Nutritional assessment in NHANES is described with a focus on dietary data collection, analysis, and uses in nutrition monitoring [7].

## MATERIALS AND METHODS

### Study population

The National Health and Nutrition Examination Survey (NHANES, National Health and Nutrition Examination Survey) is a population-based cross-sectional survey designed to collect information about the United States. Information on the health and nutrition of the country’s family population. The project surveys a nationally representative sample each year, and these populations are located in counties across the country. NHANES interviews include demographics, socioeconomics, diet and health related issues. The physical examination part includes physiological measurements, laboratory examinations, etc. [8].

**Figure 1:**
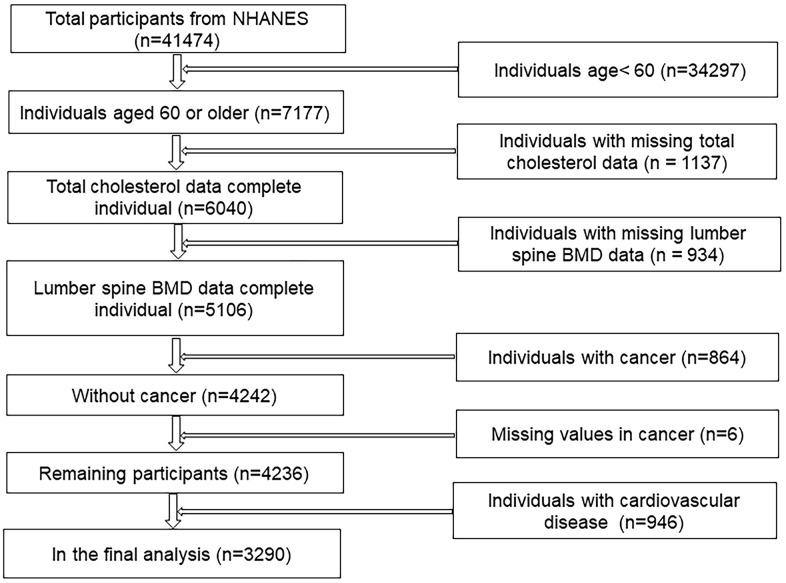
Flow chart of sample selection from the NHANES.

Our analysis is based on 1999-2006 data, which represents the three cycles of NHANES. The specific exclusion process is shown in the screening flowchart (Figure 1). In the end, a total of 3290 cancer-free participants over 60 years of age were included in our analysis.

### Variables

The exposure variable for this study was serum total cholesterol. In the mobile examination center (MEC) laboratory, blood specimens are processed, stored, and shipped to the Johns Hopkins University Lipoprotein Analytical Laboratory for analysis. The outcome variable was lumbar spine BMD, measured by DEXA. The following categorical variables were included as covariates in our analysis: age, sex, race / ethnicity, physical activity. Continuous covariates were included in our analysis: income poverty rate, blood urea nitrogen (BUN), total protein, serum uric acid, blood calcium and body mass index (BMI). Detailed information on total cholesterol, lumbar spine BMD, and covariates is available in http://www.cdc.gov/nchs/nhanes/ Available publicly.

### Statistical data analysis

Data were analyzed with the use of the statistical packages R (The R Foundation; http://www.r-project.org; version 3.6.3) and EmpowerStats (https://www.empowerstats.net, X&Y solutions, Inc. Boston, MA). And P values < 0.05 were considered statistically significant. We performed weighted and variance estimation analyses to account for significant variance in our dataset. Weighted multiple logistic regression models were used to assess the association between total cholesterol and BMD of the lumbar spine. We calculated differences between groups using a weighted chi square test for categorical variables or a weighted linear regression model for continuous variables. Subgroup analysis was performed by stratified multiple regression analysis. In addition, smooth curve fitting and generalized additive models were used to address the nonlinear relationship between total cholesterol and lumbar spine BMD. For non-linearity in the model, a recursive algorithm was used to calculate the inflection point in the relationship of total cholesterol and BMD when non-linearity was detected, with a bi segmented linear regression model on either side of the inflection point.

### Data availability statement

All data and materials were sourced from public databases. The datasets for this study can be found at https://www.cdc.gov/nchs/nhanes/.

## RESULTS

A total of 3290 participants aged 60-85 were included in our analysis. The weighted characteristics of the participants were subdivided according to the tertiles of serum total cholesterol (low: ≥75 mg/dL to 191 mg/dL; medium: ≥ 192 mg/dL to <224 mg/dL; High: ≥225 mg/dL to <704 mg/dL). As shown in Table 1, there were significant differences in baseline characteristics between the tertiles of total cholesterol except for race / ethnicity. Compared with other subgroups, participants in the highest tertile of total cholesterol were more likely to be female, 60-69 years old, and sedentary. Participants in the top tertile of total cholesterol had lower income to poverty ratio, blood urea nitrogen, serum uric acid, and body mass index levels but higher total protein and serum calcium levels.

**Table 1:** Baseline characteristics of participants (N=3290).

**Total cholesterol (mg/dL)**	**Total**	**Low (≥75 to 191)**	**Middle (≥192 to <224)**	**High (≥225 to <704)**	***P*-value**
**Sex (%)**					<0.001
**Male**	50.608	64.537	50.229	37.534	
**Female**	49.392	35.463	49.771	62.466	
**Age (%)**					0.034
**60-69 years**	52.219	49.259	52.521	54.781	
**>= 70 years**	47.781	50.741	47.479	45.219	
**Race/ethnicity (%)**					0.167
**Non-Hispanic White**	76.869	74.537	79.560	76.497	
**Non-Hispanic Black**	16.687	18.241	15.032	16.801	
**Other Hispanic**	3.404	3.611	2.750	3.843	
**Other races - Including multi-racial**	3.040	3.611	2.658	2.860	
**Physical activity (%)**					0.074
**Sedentary**	33.818	34.394	31.938	35.094	
**Low**	25.962	26.938	24.440	26.509	
**Moderate**	15.228	14.115	15.482	16.038	
**High**	24.992	24.553	28.140	22.358	
**Income to poverty ratio**	2.443 ± 1.497	2.489 ± 1.502	2.498 ± 1.520	2.345 ± 1.466	0.038
**Blood urea nitrogen (mg/dL)**	16.410 ± 7.092	16.957 ± 8.111	16.091 ± 6.668	16.091 ± 6.668	0.008
**Total protein (mg/dL)**	7.316 ± 0.516	7.251 ± 0.527	7.338 ± 0.511	7.359 ± 0.504	<0.001
**Serum uric acid (mg/dL)**	5.656 ± 1.460	5.787 ± 1.511	5.619 ± 1.439	5.565 ± 1.421	0.001
**Serum calcium (mg/dL)**	9.469 ± 0.413	9.410 ± 0.418	9.460 ± 0.397	9.533 ± 0.415	<0.001
**Body mass index**	28.343 ± 5.459	28.711 ± 5.816	28.045 ± 5.156	28.280 ± 5.376	0.017
**Lumber spine BMD (g/cm^2^)**	1.014 ± 0.195	1.044 ± 0.204	1.044 ± 0.204	0.982 ± 0.181	<0.001

Note: Mean +/- standard deviation for: age, income to poverty, ratio blood urea nitrogen, total protein, serum uric acid, serum calcium, BMI, total days drink year, total BMD, head BMD, lumber spine BMD, lumber pelvis BMD. P value was calculated using weighted linear regression model. % For: age, sex, race/ethnicity, education, physical activity. P value was calculated using weighted chi-square test. Weighted by: Full sample mobile examination center exam weight.

BMD, Bone mineral density; BMI, Body mass index.

The results of multiple regression equation analysis are shown in Table 2. In the unadjusted model, total cholesterol was negatively correlated with lumbar spine BMD (β=-0.0007, 95% CI: -0.0008, -0.0005, P<0.0001). After adjusting for confounding factors, this positive correlation still exists in Model 2 (β=-0.0004, 95% CI: -0.0005, -0.0002, P<0.0001) and Model 3(β=-0.0002, 95% CI: -0.0004, -0.0001, P=0.0034), P for trend is less than 0.001. We also stratified by continuous variables (third quantiles) and categorical variables; The results are shown in Table 2.

**Table 2:** Relationship between total cholesterol and lumbar spine bone mineral density.

**Outcome**	**Crude model**			**Model I**			**Model II**	
	**β (95%CI)**	**P-value**		**β (95%CI)**	**P-value**		**β (95%CI)**	**P-value**
**Total cholesterol (10 mg/dL)**	-0.007 (-0.008, -0.005)	<0.0001		-0.004 (-0.005, -0.002)	<0.0001		-0.002 (-0.004, -0.001)	0.0034
**Total cholesterol (tertiles)**								
**Low**	Reference			Reference			Reference	
**Middle**	-0.237 (-0.392, -0.082)	0.0027		-0.047 (-0.195, 0.100)	0.5293		0.012 (-0.143, 0.167)	0.8818
**High**	-0.678 (-0.831, -0.525)	<0.0001		-0.340 (-0.490, -0.191)	<0.0001		-0.184 (-0.340, -0.028)	0.0210
**P for trend**	<0.001			<0.001			0.020	
**Sex**								
**Male**	-0.004 (-0.006, -0.002)	0.0002		-0.004 (-0.006, -0.002)	0.0005		-0.003 (-0.005, -0.001)	0.0058
**Female**	-0.003 (-0.005, -0.001)	0.0026		-0.003 (-0.005, -0.001)	0.0028		-0.001 (-0.003, -0.001)	0.3472
**Age**								
**60-69 years**	-0.005 (-0.007, -0.003)	<0.0001		-0.003 (-0.005, -0.001)	0.0018		-0.002 (-0.004, 0.000)	0.1152
**>= 70 years**	-0.009 (-0.011, -0.007)	<0.0001		-0.004 (-0.006, -0.002)	0.0006		-0.003 (-0.005, -0.001)	0.0115
**Race/ethnicity**								
**Non-Hispanic White**	-0.007 (-0.009, -0.006)	<0.0001		-0.004 (-0.006, -0.002)	<0.0001		-0.003 (-0.005, -0.001)	0.0007
**Non-Hispanic Black**	-0.003 (-0.007, -0.001)	0.1439		-0.000 (-0.005, 0.004)	0.8174		-0.000 (-0.004, 0.004)	0.9697
**Other Hispanic**	-0.001 (-0.009, 0.007)	0.7496		-0.000 (-0.008, 0.008)	0.9834		0.005 (-0.003, 0.014)	0.2047
**Other races - Including multi-racial**	-0.003 (-0.012, 0.005)	0.4869		-0.002 (-0.010, 0.007)	0.7036		-0.001 (-0.010, 0.008)	0.8562
**Serum uric acid (tertiles)**								
**Low**	-0.007 (-0.010, -0.004)	<0.0001		-0.004 (-0.007, -0.002)	0.0004		-0.003 (-0.006, -0.001)	0.0150
**Middle**	-0.006 (-0.008, -0.003)	<0.0001		-0.004 (-0.006, -0.001)	0.0074		-0.003 (-0.005, -0.000)	0.0452
**High**	-0.006 (-0.008, -0.003)	<0.0001		-0.003 (-0.005, -0.000)	0.0250		-0.001 (-0.003, 0.002)	0.4791
**Physical activity (tertiles)**								
**Sedentary**	-0.007 (-0.010, -0.004)	<0.0001		-0.004 (-0.007, -0.001)	0.0025		-0.003 (-0.005, -0.000)	0.0729
**Low**	-0.006 (-0.009, -0.003)	<0.0001		-0.003 (-0.006, -0.001)	0.0125		-0.001 (-0.003, 0.002)	0.7098
**Moderate**	-0.010 (-0.015, -0.006)	<0.0001		-0.006 (-0.010, -0.002)	0.0080		-0.004 (-0.008, -0.000)	0.0769
**High**	-0.005 (-0.008, -0.002)	0.0010		-0.003 (-0.006, -0.000)	0.0573		-0.002 (-0.005, 0.001)	0.1684
**Income to poverty ratio (tertiles)**								
**Low**	-0.003 (-0.005, -0.001)	0.0115		-0.001 (-0.004, -0.001)	0.2478		-0.000 (-0.002, 0.002)	0.8079
**Middle**	-0.007 (-0.010, -0.005)	<0.0001		-0.004 (-0.007, -0.001)	0.0032		-0.003 (-0.005, -0.000)	0.0642
**High**	-0.008 (-0.0011, -0.005)	<0.0001		-0.005 (-0.007, -0.002)	0.0017		-0.004 (-0.007, -0.001)	0.01591
**Blood urea nitrogen (tertiles)**								
**Low**	-0.005 (-0.008, -0.002)	0.0003		-0.003 (-0.005, 0.000)	0.0610		-0.002 (-0.005, 0.001)	0.2595
**Middle**	-0.007 (-0.010, -0.004)	<0.0001		-0.003 (-0.006, -0.001)	0.0148		-0.002 (-0.005, -0.001)	0.1428
**High**	-0.008 (-0.010, -0.005)	<0.0001		-0.005 (-0.007, -0.002)	<0.0001		-0.003 (-0.005, -0.000)	0.0181
**Total protein (tertiles)**								
**Low**	-0.006 (-0.009, -0.003)	<0.0001		-0.003 (-0.006, -0.001)	0.0081		-0.002 (-0.004, 0.001)	0.1597
**Middle**	-0.006 (-0.008, -0.003)	<0.0001		-0.003 (-0.006, -0.000)	0.0280		-0.002 (-0.005, -0.000)	0.1077
**High**	-0.008 (-0.010, -0.005)	<0.0001		-0.004 (-0.006, -0.001)	0.0035		-0.002 (-0.005, -0.000)	0.0539
**Serum calcium (tertiles)**								
**Low**	-0.008 (-0.011, -0.005)	<0.0001		-0.005 (-0.008, -0.002)	0.0011		-0.005 (-0.008, -0.002)	0.0033
**Middle**	-0.005 (-0.007, -0.002)	0.0003		-0.002 (-0.004, 0.001)	0.2133		-0.001 (-0.003, 0.002)	0.4794
**High**	-0.007 (-0.009, -0.004)	<0.0001		-0.004 (-0.006, -0.002)	0.0004		-0.003 (-0.005, -0.000)	0.0390
**BMI (tertiles)**								
**Low**	-0.006 (-0.009, -0.004)	<0.0001		-0.003 (-0.006, -0.001)	0.072		-0.004 (-0.006, -0.001)	0.0053
**Middle**	-0.005 (-0.008, -0.003)	<0.0001		-0.002 (-0.004, 0.000)	0.904		-0.000 (-0.003, 0.002)	0.8110
**High**	-0.007 (-0.010, -0.005)	<0.0001		-0.004 (-0.006, -0.001)	0.016		-0.004 (-0.007, -0.002)	0.0007

Outcome variable: Total BMD. Exposure variable: Total cholesterol.

The crude model is not adjusted. Model I adjusted for age, sex, and serum uric acid. Model II adjusted for age, sex, serum uric acid, race ethnicity, education, income to poverty ratio, blood urea nitrogen, total protein, serum uric acid, serum calcium, physical activity, BMI.

Abbreviations: CI, confidence interval; BMD, bone mineral density BMI, body mass index.

Table 3 is a stratified analysis of total cholesterol versus lumbar spine bone mineral density. Except for Non-Hispanic Black and Other Hispanic in race/ethnicity, with the lowest tertile of total cholesterol as the reference, the other items of β in the highest tertile of total cholesterol were negative, and the P was less than 0.01, and the difference was statistically significant.

**Table 3:** Stratified analysis between total cholesterol and lumbar bone mineral density.

	**Total cholesterol (tertiles)**		
	**Low**	**Middle β (95%CI) P value**	**High β (95%CI) P value**
**Age**			
**60-69 years**	Reference	-0.01 (-0.03, 0.01) 0.2533	-0.05 (-0.07, -0.03) <0.0001
**>= 70 years**	Reference	-0.04 (-0.06, -0.02) 0.0009	-0.10 (-0.13, -0.08) <0.0001
**Sex**			
**Male**	Reference	-0.00 (-0.02, 0.02) 0.9288	-0.01 (-0.03, 0.01) 0.3765
**Female**	Reference	-0.01 (-0.03, 0.01) 0.3765	-0.03 (-0.05, -0.01) 0.0021
**Race ethnicity**			
**Non-Hispanic White**	Reference	-0.02 (-0.04, -0.01) 0.0059	-0.08 (-0.10, -0.06) <0.0001
**Non-Hispanic Black**	Reference	-0.03 (-0.07, 0.02) 0.2688	-0.02 (-0.06, 0.03) 0.4768
**Other Hispanic**	Reference	-0.00 (-0.08, 0.08) 0.9424	0.02 (-0.05, 0.10) 0.5491
**Other races - Including multi-racial**	Reference	-0.05 (-0.14, 0.04) 0.2654	-0.07 (-0.15, 0.01) 0.0969
**Physical activity**			
**Sedentary**	Reference	-0.04 (-0.07, -0.01) 0.0096	-0.07 (-0.10, -0.05) <0.0001
**Low**	Reference	-0.07 (-0.10, -0.03) <0.0001	-0.08 (-0.11, -0.05) <0.0001
**Moderate**	Reference	-0.02 (-0.06, 0.03) 0.4257	-0.09 (-0.13, -0.04) <0.0001
**High**	Reference	0.01 (-0.02, 0.04) 0.6042	-0.04 (-0.07, -0.01) 0.0139
**Income to poverty ratio (tertiles)**			
**0-1.37**	Reference	-0.03 (-0.06, -0.00) 0.0432	-0.04 (-0.07, -0.02) 0.0024
**1.38-2.91**	Reference	-0.03 (-0.06, 0.00) 0.0799	-0.07 (-0.10, -0.04) <0.0001
**2.93-5.00**	Reference	-0.03 (-0.05, -0.00) 0.0475	-0.07 (-0.10, -0.04) <0.0001
**Blood urea nitrogen (tertiles, mg/dL)**			
**2.00 - 12.00**	Reference	-0.04 (-0.06, -0.01) 0.0147	-0.05 (-0.08, -0.02) 0.0003
**13.00 - 16.00**	Reference	-0.01 (-0.03, 0.02) 0.6859	-0.06 (-0.09, -0.03) <0.0001
**17.00 - 98.00**	Reference	-0.03 (-0.06, -0.01) 0.0120	-0.09 (-0.11, -0.06) <0.0001
**Total protein (tertiles, mg/dL)**			
**5.40 - 7.00**	Reference	-0.03 (-0.05, 0.00) 0.0764	-0.07 (-0.10, -0.04) <0.0001
**7.10 - 7.40**	Reference	-0.03 (-0.05, 0.00) 0.0509	-0.05 (-0.07, -0.02) 0.0002
**7.50 - 11.00**	Reference	-0.02 (-0.05, 0.01) 0.1544	-0.08 (-0.11, -0.05) <0.0001
**Serum uric acid (tertiles, mg/dL)**			
**1.50 - 4.80**	Reference	-0.00 (-0.03, 0.02) 0.7715	-0.07 (-0.09, -0.04) <0.0001
**4.90 - 6.00**	Reference	-0.01 (-0.03, 0.02) 0.6510	-0.04 (-0.07, -0.01) 0.0051
**6.10 - 13.70**	Reference	-0.04 (-0.07, -0.02) 0.0016	-0.07 (-0.10, -0.05) <0.0001
**Serum calcium (tertiles, mg/dL)**			
**6.70 - 9.20**	Reference	-0.05 (-0.08, -0.02) 0.0014	-0.07 (-0.10, -0.04) <0.0001
**9.30 - 9.50**	Reference	0.00 (-0.03, 0.03) 0.9714	-0.07 (-0.09, -0.04) <0.0001
**9.60 – 11.30**	Reference	-0.02 (-0.05, 0.00) 0.0913	-0.06 (-0.08, -0.03) <0.0001
**Body mass index (tertiles)**			
**15.18 - 25.72**	Reference	-0.00 (-0.03, 0.02) 0.7332	-0.06 (-0.09, -0.03) <0.0001
**25.73 – 29.86**	Reference	-0.00 (-0.03, 0.02) 0.6066	-0.06 (-0.09, -0.04) <0.0001
**29.87 – 57.31**	Reference	-0.03 (-0.06, -0.01) 0.0111	-0.06 (-0.09, -0.04) <0.0001

We performed a univariate analysis of lumbar bone mineral density (Supplementary Table 1). Compared with men, women’s lumbar spine BMD is reduced by 0.119, which is a relatively large difference, and the difference is statistically significant (P value less than 0.0001). Other results are shown in Supplementary Table 1.

**Figure 2:**
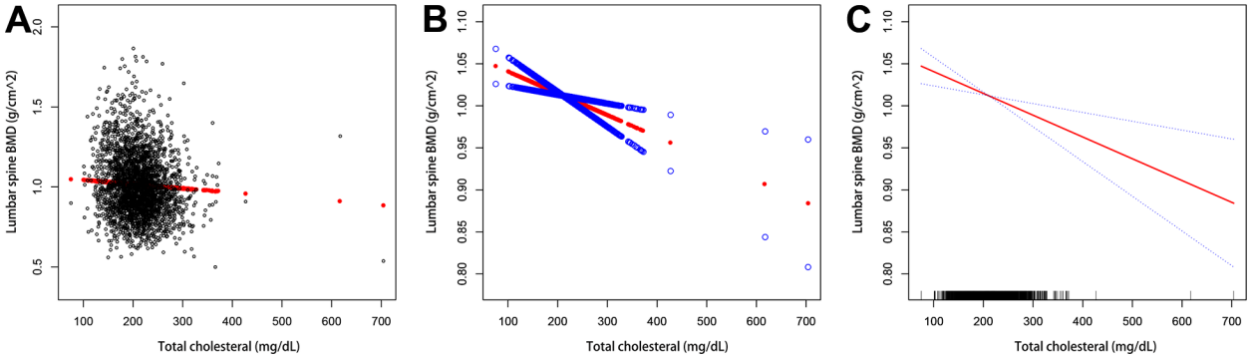
**The association between serum total cholesterol and lumbar bone mineral density.** (**A**) Each black dot represents a sample. (**B**, **C**) The solid arc line represents the smooth curve fit between the variables. The blue bar represents the 95% confidence interval of the fit. Adjustments were made for age, gender, race/ethnicity, physical activity, income poverty rate, blood urea nitrogen, serum urea, total protein, blood phosphorus, and blood calcium.

**Figure 3:**
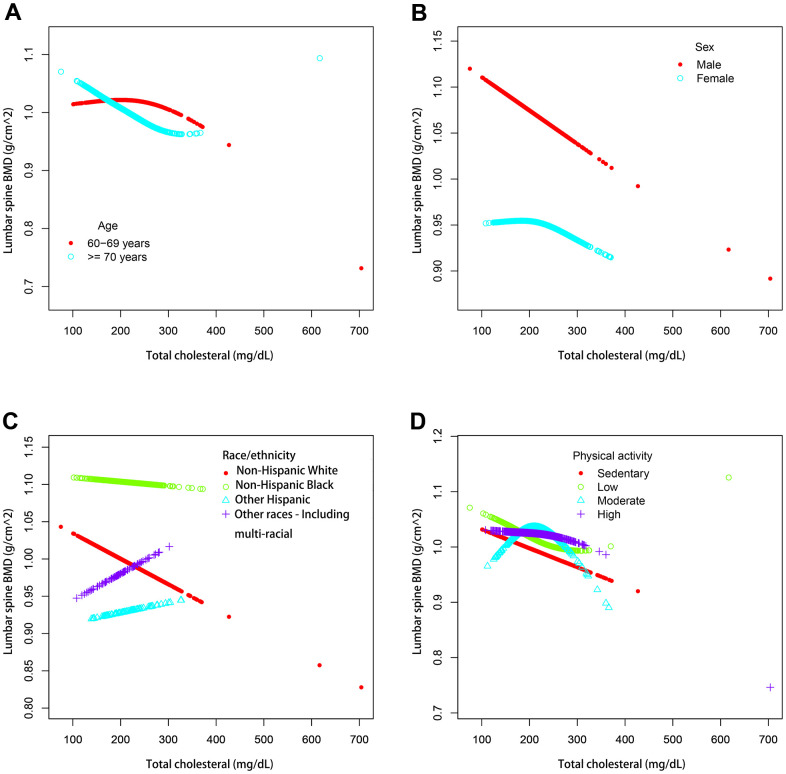
**The association between serum total cholesterol and lumbar spine BMD stratified by different categorical variables.** (**A**) Stratified by age. Sex, race / ethnicity, physical activity, income poverty rate, blood urea nitrogen, total protein, serum uric acid, blood calcium and body mass index were adjusted. (**B**) Stratified by sex. Age, race / ethnicity, physical activity, income poverty rate, blood urea nitrogen, total protein, serum uric acid, blood calcium and body mass index were adjusted. (**C**) Stratified by race / ethnicity. Age, sex, physical activity, income poverty rate, blood urea nitrogen, total protein, serum uric acid, blood calcium and body mass index were adjusted. (**D**) Stratified by physical activity. Age, sex, race / ethnicity, income poverty rate, blood urea nitrogen, total protein, total protein, serum uric acid, blood calcium and body mass index were adjusted.

**Figure 4:**
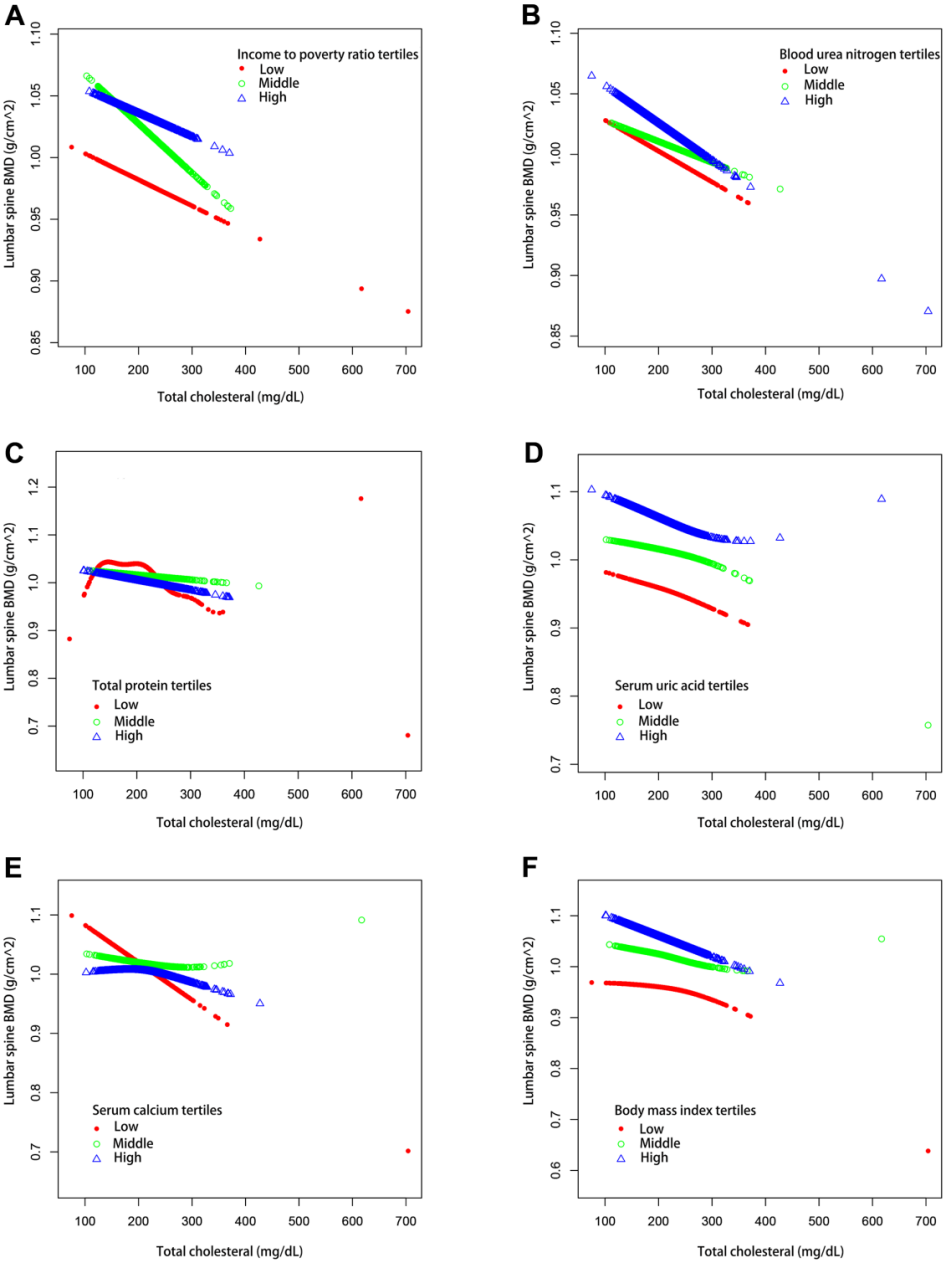
**The association between serum total cholesterol and lumbar spine BMD stratified by tertiles of different continuous variables.** (**A**) Stratified by income poverty rate tertiles. Age, Sex, race / ethnicity, physical activity, blood urea nitrogen, total protein, serum uric acid, blood calcium and body mass index were adjusted. (**B**) Stratified by blood urea nitrogen tertiles. Age, Sex, race / ethnicity, physical activity, income poverty rate, total protein, serum uric acid, blood calcium and body mass index were adjusted. (**C**) Stratified by total protein tertiles. Age, Sex, race / ethnicity, physical activity, income poverty rate, blood urea nitrogen, serum uric acid, blood calcium and body mass index were adjusted. (**D**) Stratified by serum uric acid tertiles. Age, Sex, race / ethnicity, physical activity, income poverty rate, blood urea nitrogen, total protein, blood calcium and body mass index were adjusted. (**E**) Stratified by blood calcium tertiles. Age, Sex, race / ethnicity, physical activity, income poverty rate, blood urea nitrogen, total protein, serum uric acid and body mass index were adjusted. (**F**) Stratified by body mass index tertiles. Age, Sex, race / ethnicity, physical activity, income poverty rate, blood urea nitrogen, total protein, serum uric acid and blood calcium were adjusted.

We also performed weighted generalized additive models and smoothing curve fitting to assess their association (Figure 2). Figure 2 shows that total cholesterol is linearly negatively correlated with lumbar bone mineral density. We also performed smooth curve fitting in subgroups stratified by categorical variables (Figure 3). Figure 3A shows that there is a turning point in the fitting curve for elderly people older than or equal to 70 years old, at a total cholesterol of 280 mg/dL (Supplementary Table 2). Figure 3D shows that the fitting curve of older people with more than moderate physical activity has a turning point, at a total cholesterol of 199 mg/dL (Supplementary Table 2). Other turning points are shown in Figure 4 and Supplementary Table 2.

## DISCUSSION

Samelson, Cupples [9] believes that the cholesterol levels of women and men from young adult to middle-aged do not seem to have long-term clinical significance for later osteoporosis. However, Zolfaroli, Ortiz [5]’s research suggests that there is a positive correlation between cholesterol and bone mineral density in the lumbar spine and femoral neck in postmenopausal women. Some researchers also found a moderate negative correlation between BMD and serum cholesterol levels [1]. Therefore, the relationship between bone mineral density and serum total cholesterol level is complex and unclear.

The primary objective of this study was to investigate whether total cholesterol was independently associated with lumbar spine BMD. In this study, we used a nationally representative sample of older Americans without cancer (n=3290). Our results suggest a significant inverse association between serum cholesterol levels and bone mineral density of the lumbar spine in US non cancer affected older adults aged 60 years or older. Older adults ≥ 70 years of age had an inflection point at 280 mg / dl, log likelihood ratio tests was 0.002. And those with moderate physical activity had an inflection point at 199 mg / dl, the p value from the log likelihood ratio test was 0.005. There was an inflection point at 277 mg / dl for older adults in the highest tertile of serum uric acid (the p value from the log likelihood ratio test was 0.012) and at 275 mg / dl for older adults in the middle tertile of serum calcium (the p value from the log likelihood ratio test was 0.023). The smooth curves they fitted were all U-shaped. Their fitted smooth curves were all U-shaped, all are statistically significant.

The exact mechanism between total cholesterol and bone metabolism is unclear, the correlation between total cholesterol and bone mineral density remains controversial [10–12]. There may be the following reasons. Bones are highly innervated and vascularized. The seemingly closed system of the skeleton, tightly linked to systemic metabolic homeostasis, is dynamically regulated by hormones and nutrients. A large number of epidemiological studies have demonstrated a positive association between the risk of cardiovascular disease and osteoporosis [13–16]. Bone metabolism is a continuous cycle of bone formation and resorption, which is coordinated by osteoblasts, osteocytes, and osteoclasts.

Free cholesterol may inhibit BMP2, thereby blocking Runx2, Alpl, and COL1A1 expression in osteoblasts and subsequently inhibiting osteoblast differentiation [12, 17]. Cholesterol and its metabolites influence the bone homeostasis through modulating the differentiation and activation of osteoblasts and osteoclasts [12]. It has been shown that inhibition of cholesterol biosynthesis inhibits mRNA expression in the precursor stromal bone marrow cells of osteoblasts, thereby preventing osteogenic differentiation and achieving improved BMD [10, 18, 19]. Treatment of rodents with the cholesterol lowering drugs simvastatin and lovastatin both increases bone formation [20]. In an animal study, a high cholesterol diet showed a decrease in femoral BMD accompanied by an increase in serum OCN and carboxy terminal collagen cross-linking (CTX), suggesting that high cholesterol may increase bone turnover [17, 20]. High fat diet also induced cathepsin K-Positive osteoclasts and RANKL expression, leading to enhanced osteoclastogenesis, resulting in decreased bone mineral density [21]. Mazidi et al. [22] found that cholesterol levels increased indicators of inflammation in humans, and, in a study by Wang et al. found that inflammatory factors decreased BMD by affecting osteoclast activation or function [23], therefore, high cholesterol levels may affect BMD by activating the inflammatory response.

In this study, bone mineral density levels in older adults were the outcome variable and serum cholesterol levels were the exposure variable. After adjusting for more than a dozen variables such as age, sex, and race, we found that high serum cholesterol levels were an independent risk factor for reduced BMD levels in older adults.

In this study, we analyzed a representative sample of a multi-ethnic population to better generalize the American population. This large sample size allows us to perform further subgroup analysis. This is the main advantage of this research. Our findings suggest that older adults should be told that cholesterol is controlled at an appropriate level and that older patients with hypercholesterolemia have reduced bone mineral density.

Our study still has some deficiencies. First, other confounding factors not included in this study may have had an impact on the results. Second, because this study used a cross-sectional design, it is difficult to determine whether there is a causal relationship between total cholesterol and lumbar spine BMD. Therefore, the role of total cholesterol in bone metabolism requires further studies with large samples.

## CONCLUSIONS

There is a negative association between total cholesterol and lumbar spine BMD in non-cancer elderly greater than or equal to 60 years of age.

## Supplementary Material

Supplementary Table 1

Supplementary Table 2
