# Supplementary material for: Associations between serum total cholesterol level and bone mineral density in older adults
Source: Aging (Albany NY). 2023 Feb 10;15(5):1330–42. doi: 10.18632/aging.204514 (PMC10042689; doi:10.18632/aging.204514)
Supplement: Supplementary Table 1 [file aging-15-204514-s001.pdf]

SUPPLEMENTARY TABLE

Supplementary Table 1. Univariate analysis for lumbar spine bone mineral density.

	Statistics	β (95%CI)	*P*-value
**Total cholesterol**	210.443 ± 42.618	0.001 (-0.001, -0.001)	<0.0001
**Total cholesterol quartiles**			
**Low**	1080 (32.827%)	Reference	
**Middle**	1091 (33.161%)	-0.024 (-0.039, -0.008)	0.0028
**High**	1119 (34.012%)	-0.068 (-0.083, -0.053)	<0.0001
**Sex**			
**Male**	1665 (50.608%)	Reference	
**Female**	1625 (49.392%)	-0.119 (-0.131, -0.107)	<0.0001
**Race/ethnicity**			
**Non-Hispanic white**	2529 (76.869%)	Reference	
**Non-Hispanic black**	549 (16.687%)	0.088 (0.065, 0.111)	<0.0001
**Other Hispanic**	112 (3.404%)	-0.069 (-0.101, -0.038)	<0.0001
**Other races - Including multi-racial**	100 (3.040%)	-0.032 (-0.063, -0.002)	0.0365
**Age**			
**60-69 years**	1718 (52.219%)	Reference	
**>= 70 years**	1572 (47.781%)	-0.020 (-0.033, -0.007)	0.0020
**Income to poverty ratio**	2.443 ± 1.497	0.015 (0.011, 0.020)	<0.0001
**Income to poverty ratio (tertile)**			
**Low**	993 (33.311%)	Reference	
**Middle**	986 (33.076%)	0.051 (0.033, 0.069)	<0.0001
**High**	1002 (33.613%)	0.065 (0.048, 0.081)	<0.0001
**Blood urea nitrogen**	16.410 ± 7.092	0.002 (0.001, 0.003)	<0.0001
**Blood urea nitrogen (tertile)**			
**Low**	900 (27.447%)	Reference	
**Middle**	1052 (32.083%)	0.017 (0.001, 0.034)	0.0386
**High**	1327 (40.470%)	0.029 (0.013, 0.044)	0.0004
**Total protein**	7.316 ± 0.516	-0.013 (-0.025, 0.000)	0.0523
**Total protein (tertile)**			
**Low**	978 (29.835%)	Reference	
**Middle**	1066 (32.520%)	-0.017 (-0.033, -0.002)	0.0258
**High**	1234 (37.645%)	-0.019 (-0.034, -0.003)	0.0167
**Serum uric acid**	5.656 ± 1.460	0.030 (0.026, 0.035)	<0.0001
**Serum uric acid (tertile)**			
**Low**	0.030 (0.026, 0.035)	Reference	
**Middle**	1102 (33.608%)	0.062 (0.047, 0.078)	<0.0001
**High**	1171 (35.712%)	0.100 (0.085, 0.115)	<0.0001
**Serum calcium**	9.469 ± 0.413	-0.044 (-0.060, -0.028)	<0.0001
**Serum calcium (tertile)**			
**Low**	935 (28.515%)	Reference	
**Middle**	1020 (31.107%)	-0.007 (-0.023, 0.010)	0.4448
**High**	1324 (40.378%)	-0.035 (-0.050, -0.019)	<0.0001
**Physical activity**			
**Sedentary**	1046 (33.818%)	Reference	
**Low**	803 (25.962%)	0.018 (0.000, 0.035)	0.0464

**Moderate**	471 (15.228%)	0.034 (0.014, 0.054)	0.0009
**High**	773 (24.992%)	0.033 (0.016, 0.050)	0.0002
**BMI**	28.343 ± 5.459	0.010 (0.009, 0.011)	<0.0001
**BMI (tertile)**			
**Low**	1074 (33.344%)	Reference	
**Middle**	1073 (33.313%)	0.066 (0.051, 0.081)	<0.0001
**High**	1074 (33.344%)	0.115 (0.100, 0.130)	<0.0001

Abbreviations: CI, confidence interval; BMI, Body mass index.
