# Supplementary material for: Associations between serum total cholesterol level and bone mineral density in older adults
Source: Aging (Albany NY). 2023 Feb 10;15(5):1330–42. doi: 10.18632/aging.204514 (PMC10042689; doi:10.18632/aging.204514)
Supplement: Supplementary Table 2 [file aging-15-204514-s002.docx]

**Supplementary Table 2. Threshold effect and saturation effect analysis.**

|  | **Model I** |  | **Model II** | | | | | |
| --- | --- | --- | --- | --- | --- | --- | --- | --- |
|  | **A straight-line effect** |  | **Fold points (K)** | **< K-segment effect 1** | **>K-segment Effect 2** | **Effect size difference of 2 versus 1** | **Equation predicted values at break points** | **Log likelihood ratio tests** |
| **Total** |  |  |  |  |  |  |  |  |
| **Adjusted β**  **(95% CI)** | -0.0002  (-0.0004, -0.0001) |  | 146 | 0.0009  (-0.0008, 0.0026) | -0.0003  (-0.0004, -0.0001) | -0.0012  (-0.0029, 0.0005) | 1.0580  (1.0443, 1.0717) | 0.179 |
| ***P*-value** | 0.0034 |  |  | 0.2841 | 0.0013 | 0.1801 |  |  |
| **Stratification variables** | |  |  |  |  |  |  |  |
| ***Age*** |  |  |  |  |  |  |  |  |
| **60-69 years** |  |  |  |  |  |  |  |  |
| **Adjusted β**  **(95% CI)** | -0.0002  (-0.0004, -0.0000) |  | 249 | 0.0001  (-0.0002, 0.0004) | -0.0009  (-0.0014, -0.0003) | -0.0010  (-0.0016, -0.0003) | 1.0120  (0.9966, 1.0274) | 0.004 |
| ***P*-value** | 0.1152 |  |  | 0.4724 | 0.0011 | 0.0041 |  |  |
| **>= 70 years** |  |  |  |  |  |  |  |  |
| **Adjusted β**  **(95% CI)** | -0.0003  (-0.0005, -0.0001) |  | 280 | -0.0005  (-0.0008, -0.0002) | 0.0007  (-0.0000, 0.0014) | 0.0012  (0.0004, 0.0020) | 0.9040  (0.8812, 0.9267) | 0.002 |
| ***P*-value** | 0.0115 |  |  | 0.0002 | 0.0431 | 0.0025 |  |  |
| ***Sex*** |  |  |  |  |  |  |  |  |
| **Male** |  |  |  |  |  |  |  |  |
| **Adjusted β**  **(95% CI)** | -0.0003  (-0.0005, -0.0001) |  | 203 | 0.0000  (-0.0005, 0.0004) | -0.0005  (-0.0008, -0.0001) | -0.0005  (-0.0011, 0.0002) | 1.0827  (1.0680, 1.0974) | 0.166 |
| ***P*-value** | 0.0058 |  |  | 0.9007 | <0.0047 | 0.1682 |  |  |
|  |  |  |  |  |  |  |  |  |

**Continued table 5.**

|  | **Model I** |  | **Model II** | | | | | |
| --- | --- | --- | --- | --- | --- | --- | --- | --- |
|  | **A straight-line effect** |  | **Fold points (K)** | **< K-segment effect 1** | **> K-segment Effect 2** | **Effect size difference of 2 versus 1** | **Equation predicted values at break points** | **Log likelihood ratio tests** |
| **Female** |  |  |  |  |  |  |  |  |
| **Adjusted β**  **(95% CI)** | -0.0001  (-0.0003, 0.0001) |  | 208 | 0.0003  (-0.0003, 0.0008) | -0.0003  (-0.0006, -0.0000) | -0.0005  (-0.0012, -0.0002) | 0.9619  (0.9477, 0.9762) | 0.141 |
| ***P*-value** | 0. 3472 |  |  | 0.3341 | 0.0849 | 0.1432 |  |  |
| ***Race/ethnicity*** |  |  |  |  |  |  |  |  |
| **Non-Hispanic White** |  |  |  |  |  |  |  |  |
| **Adjusted β**  **(95% CI)** | -0.0003  (-0.0005, -0.0001) |  | 146 | 0.00013  (-0.0007, 0.0032) | -0.0003  (-0.0005, -0.0002) | -0.0016  (-0.0036, 0.0004) | 1.0611  (1.0459, 1.0763) | 0.111 |
| ***P*-value** | 0.0007 |  |  | 0.1984 | 0.0002 | 0.1124 |  |  |
| **Non-Hispanic Black** |  |  |  |  |  |  |  |  |
| **Adjusted β**  **(95% CI)** | -0.0000  (-0.0004, 0.0004) |  | 270 | -0.0001  (-0.0004, 0.0006) | -0.0007  (-0.0027, 0.0013) | 0.0008  (-0.0030, 0.0014) | 1.0832  (1.0432, 1.1232) | 0.454 |
| ***P*-value** | 0.9697 |  |  | 0.7356 | 0.4676 | 0.4617 |  |  |
| **Other Hispanic** |  |  |  |  |  |  |  |  |
| **Adjusted β**  **(95% CI)** | 0.0005  (-0.0003, 0.0014) |  | 161 | -0.0093  (-0.0187, 0.0001) | 0.0008  (-0.0000, 0.0016) | 0.0101  (0.0005, 0.0197) | 0.9200  (0.8567, 0.9833) | 0.026 |
| ***P*-value** | 0.2047 |  |  | 0.0556 | 0.0661 | 0.0425 |  |  |

**Continued table 5.**

|  | **Model I** |  | **Model II** | | | | | |
| --- | --- | --- | --- | --- | --- | --- | --- | --- |
|  | **A straight-line effect** |  | **Fold points (K)** | **< K-segment effect 1** | **>K-segment Effect 2** | **Effect size difference of 2 versus 1** | **Equation predicted values at break points** | **Log likelihood ratio tests** |
| **Other races - Including multi-racial** |  |  |  |  |  |  |  |  |
| **Adjusted β**  **(95% CI)** | -0.0001  (-0.0010, 0.0008) |  | 273 | -0.0004  (-0.0014, 0.0006) | -0.0113  (-0.0040, 0.0265) | 0.0117  (-0.0040, 0.0274) | 0.9406  (0.8575, 1.0237) | 0.112 |
| ***P*-value** | 0.8562 |  |  | 0.4014 | 0.1510 | 0.1474 |  |  |
| ***Physical activity*** |  |  |  |  |  |  |  |  |
| **Sedentary** |  |  |  |  |  |  |  |  |
| **Adjusted β**  **(95% CI)** | -0.0003  (-0.0005, -0.0000) |  | 146 | 0.0008  (-0.0020, 0.0035) | -0.0003  (-0.0006, -0.0000) | -0.0010  (-0.0039, 0.0018) | 1.0381  (1.0128, 1.0635) | 0.470 |
| ***P*-value** | 0.0729 |  |  | 0.5946 | 0.0537 | 0.4735 |  |  |
| **Low** |  |  |  |  |  |  |  |  |
| **Adjusted β**  **(95% CI)** | -0.0001  (-0.0003, 0.0002) |  | 273 | -0.0003  (-0.0006, 0.0001) | 0.0007  (0.0000, 0.0014) | 0.0010  (0.0001, 0.0018) | 0.9419  (0.9156, 0.9683) | 0.020 |
| ***P*-value** | 0.7098 |  |  | 0.1028 | 0.0489 | 0.0217 |  |  |
| **Moderate** |  |  |  |  |  |  |  |  |
| **Adjusted β**  **(95% CI)** | -0.0004  (-0.0008, 0.0000) |  | 199 | 0.0009  (-0.0001, 0.0019) | -0.0010  (-0.0016, -0.0004) | -0.0019  (-0.0033, -0.0006) | 1.0611  (1.0328, 1.0894) | 0.005 |
| ***P*-value** | 0.0769 |  |  | 0.0665 | 0.0012 | 0.0054 |  |  |

**Continued table 5.**

|  | **Model I** |  | **Model II** | | | | | |
| --- | --- | --- | --- | --- | --- | --- | --- | --- |
|  | **A straight-line effect** |  | **Fold points (K)** | **< K-segment effect 1** | **>K-segment Effect 2** | **Effect size difference of 2 versus 1** | **Equation predicted values at break points** | **Log likelihood ratio tests** |
| **High** |  |  |  |  |  |  |  |  |
| **Adjusted β**  **(95% CI)** | -0.0002  (-0.0005, 0.0001) |  | 212 | 0.0004  (-0.0001, 0.0010) | -0.0007  (-0.0011, -0.0002) | -0.0011  (-0.0020, 0.0002) | 1.0344  (1.0152, 1.0536) | 0.011 |
| ***P*-value** | 0.1684 |  |  | 0.1448 | 0.0046 | 0.0115 |  |  |
| ***Income to poverty ratio (tertiles)*** | |  |  |  |  |  |  |  |
| **Low** |  |  |  |  |  |  |  |  |
| **Adjusted β**  **(95% CI)** | -0.0000  (-0.0002, 0.0002) |  | 146 | 0.0009  (-0.0018, 0.0035) | -0.0001  (-0.0003, 0.0002) | -0.0009  (-0.0037, 0.0018) | 0.9901   (0.9680, 1.0122) | 0.493 |
| ***P*-value** | 0.8079 |  |  | 0.5110 | 0.6640 | 0.4966 |  |  |
| **Middle** |  |  |  |  |  |  |  |  |
| **Adjusted β**  **(95% CI)** | -0.0003  (-0.0005, 0.0000) |  | 147 | 0.0015  (-0.0012, 0.0042) | -0.0003  (-0.0006, -0.0000) | -0.0018  (-0.0046, 0.0010) | 1.0732  (1.0479, 1.0985) | 0.200 |
| ***P*-value** | 0.0642 |  |  | 0.2794 | 0.0276 | 0.2043 |  |  |
| **High** |  |  |  |  |  |  |  |  |
| **Adjusted β**  **(95% CI)** | -0.0004  (-0.0007, -0.0001) |  | 167 | 0.0007  (-0.0008, 0.0021) | -0.0005  (-0.0008, 0.0001) | -0.0012  (-0.0028, 0.0004) | 1.0711  (1.0506, 1.0916) | 0.152 |
| ***P*-value** | 0.0159 |  |  | 0.3639 | 0.0052 | 0.1562 |  |  |

**Continued table 5.**

|  | **Model I** |  | **Model II** | | | | | |
| --- | --- | --- | --- | --- | --- | --- | --- | --- |
|  | **A straight-line effect** |  | **Fold points (K)** | **< K-segment effect 1** | **>K-segment Effect 2** | **Effect size difference of 2 versus 1** | **Equation predicted values at break points** | **Log likelihood ratio tests** |
| **Blood urea nitrogen *(tertiles)*** | |  |  |  |  |  |  |  |
| **Low** |  |  |  |  |  |  |  |  |
| **Adjusted β**  **(95% CI)** | -0.0002  (-0.0005, -0.0001) |  | 167 | 0.0011  (-0.0003, 0.0025) | -0.0003  (-0.0007, -0.0000) | -0.0014  (-0.0030, 0.0001) | 1.0213  (0.9993, 1.0434) | 0.071 |
| ***P*-value** | 0.2595 |  |  | 0.1288 | 0.0570 | 0.0740 |  |  |
| **Middle** |  |  |  |  |  |  |  |  |
| **Adjusted β**  **(95% CI)** | -0.0002  (-0.0005, 0.0001) |  | 281 | -0.0003  (-0.0006, 0.0000) | 0.0005  (-0.0009, 0.0020) | 0.0008  (-0.0007, 0.0024) | 0.9567  (0.9300, 0.9833) | 0.292 |
| ***P*-value** | 0.1428 |  |  | 0.0755 | 0.4649 | 0.2963 |  |  |
| **High** |  |  |  |  |  |  |  |  |
| **Adjusted β**  **(95% CI)** | -0.0003  (-0.0005, -0.0000) |  | 143 | 0.0018  (-0.0012, 0.0049) | -0.0003  (-0.0006, -0.0001) | -0.0022  (-0.0053, 0.0010) | 1.0822  (1.0607, 1.1036) | 0.173 |
| ***P*-value** | 0.0181 |  |  | 0.2408 | 0.0077 | 0.1767 |  |  |
| ***Total protein (tertiles)*** | |  |  |  |  |  |  |  |
| **Low** |  |  |  |  |  |  |  |  |
| **Adjusted β**  **(95% CI)** | -0.0002  (-0.0004, 0.0001) |  | 142 | 0.0024  (-0.0003, 0.0052) | -0.0003  (-0.0005, -0.0000) | -0.0027  (-0.0056, -0.0001) | 1.0647  (1.0407, 1.0887) | 0.061 |
| ***P*-value** | 0.1597 |  |  | 0.0859 | 0.0503 | 0.0635 |  |  |

**Continued table 5.**

|  | **Model I** |  | **Model II** | | | | | |
| --- | --- | --- | --- | --- | --- | --- | --- | --- |
|  | **A straight-line effect** |  | **Fold points (K)** | **< K-segment effect 1** | **>K-segment Effect 2** | **Effect size difference of 2 versus 1** | **Equation predicted values at break points** | **Log likelihood ratio tests** |
| **Middle** |  |  |  |  |  |  |  |  |
| **Adjusted β**  **(95% CI)** | -0.0002  (-0.0005, -0.0000) |  | 281 | -0.0003  (-0.0006, 0.0000) | 0.0003  (-0.0014, 0.0021) | 0.0006  (-0.0013, 0.0025) | 0.9641  (0.9387, 0.9894) | 0.520 |
| ***P*-value** | 0.1076 |  |  | 0.0844 | 0.7060 | 0.5237 |  |  |
| **High** |  |  |  |  |  |  |  |  |
| **Adjusted β**  **(95% CI)** | -0.0002  (-0.0005, 0.0000) |  | 229 | -0.0003  (-0.0007, 0.0001) | -0.0002  (-0.0007, 0.0003) | 0.0001  (-0.0006, 0.0009) | 0.9831  (0.9646, 1.0016) | 0.718 |
| ***P*-value** | 0.0539 |  |  | 0.1600 | 0.5158 | 0.5158 |  |  |
| ***Serum uric acid (tertiles)*** | |  |  |  |  |  |  |  |
| **Low** |  |  |  |  |  |  |  |  |
| **Adjusted β**  **(95% CI)** | -0.0003  (-0.0006, -0.0001) |  | 180 | 0.0008  (-0.0002, 0.0019) | -0.0005  (-0.0009, -0.0002) | -0.0014  (-0.0026, 0.0001) | 0.9870  (0.9678, 1.0062) | 0.027 |
| ***P*-value** | 0.0150 |  |  | 0.1321 | 0.0012 | 0.0289 |  |  |
| **Middle** |  |  |  |  |  |  |  |  |
| **Adjusted β**  **(95% CI)** | -0.0003  (-0.0005, -0.0000) |  | 250 | 0.0000  (-0.0004, 0.0004) | -0.0009  (-0.0015, -0.0003) | -0.0009  (-0.0016, -0.0001) | 0.9936  (0.9728, 1.0144) | 0.029 |
| ***P*-value** | 0.0452 |  |  | 0.9927 | 0.0047 | 0.0302 |  |  |

**Continued table 5.**

|  | **Model I** |  | **Model II** | | | | | |
| --- | --- | --- | --- | --- | --- | --- | --- | --- |
|  | **A straight-line effect** |  | **Fold points (K)** | **< K-segment effect 1** | **>K-segment Effect 2** | **Effect size difference of 2 versus 1** | **Equation predicted values at break points** | **Log likelihood ratio tests** |
| **High** |  |  |  |  |  |  |  |  |
| **Adjusted β**  **(95% CI)** | -0.0001  (-0.0003, 0.0002) |  | 277 | -0.0003  (-0.0006, 0.0000) | 0.0008  (0.0001, 0.0015) | 0.0011  (0.0002, 0.0019) | 1.0021  (0.9772, 1.0270) | 0.012 |
| ***P*-value** | 0.4791 |  |  | 0.0547 | 0.0349 | 0.0123 |  |  |
| ***Serum calcium (tertiles)*** |  |  |  |  |  |  |  |  |
| **Low** |  |  |  |  |  |  |  |  |
| **Adjusted β**  **(95% CI)** | -0.0004  (-0.0007, -0.0001) |  | 272 | 0.0003  (-0.0006, 0.0001) | -0.0008  (-0.0016, -0.0001) | -0.0006  (-0.0015, 0.0004) | 0.9635  (0.9327, 0.9943) | 0.225 |
| ***P*-value** | 0.0105 |  |  | 0.1340 | 0.0340 | 0.2302 |  |  |
| **Middle** |  |  |  |  |  |  |  |  |
| **Adjusted β**  **(95% CI)** | -0.0001  (-0.0003, 0.0002) |  | 275 | -0.0003  (-0.0006, 0.0000) | 0.0006  (-0.0000, 0.0013) | 0.0009  (0.0001, 0.0017) | 0.9715  (0.9469, 0.9962) | 0.023 |
| ***P*-value** | 0.4977 |  |  | 0.0680 | 0.0642 | 0.0241 |  |  |
| **High** |  |  |  |  |  |  |  |  |
| **Adjusted β**  **(95% CI)** | -0.0002  (-0.0005, -0.0000) |  | 209 | 0.0003  (-0.0003, 0.0008) | -0.0006  (-0.0010, -0.0002) | -0.0008  (-0.0016, -0.0001) | 1.0058  (0.9891, 1.0224) | 0.034 |
| ***P*-value** | 0.0496 |  |  | 0.3165 | 0.0042 | 0.0357 |  |  |

**Continued table 5.**

|  | **Model I** |  | **Model II** | | | | | |
| --- | --- | --- | --- | --- | --- | --- | --- | --- |
|  | **A straight-line effect** |  | **Fold points (K)** | **< K-segment effect 1** | **>K-segment Effect 2** | **Effect size difference of 2 versus 1** | **Equation predicted values at break points** | **Log likelihood ratio tests** |
| **Body mass index *(tertiles)*** | |  |  |  |  |  |  |  |
| **Low** |  |  |  |  |  |  |  |  |
| **Adjusted β**  **(95% CI)** | -0.0004  (-0.0006, -0.0001) |  | 215 | 0.0003  (-0.0002, 0.0007) | -0.0008  (-0.0012, -0.0004) | -0.0011  (-0.0018, -0.0003) | 0.9567  (0.9403, 0.9731) | 0.004 |
| ***P*-value** | 0.0053 |  |  | 0.2960 | <0.0001 | 0.0045 |  |  |
| **Middle** |  |  |  |  |  |  |  |  |
| **Adjusted β**  **(95% CI)** | -0.0000  (-0.0003, 0.0002) |  | 273 | -0.0002  (-0.0005, 0.0001) | 0.0005  (-0.0002, 0.0013) | 0.0007  (-0.0002, 0.0016) | 0.9779  (0.9538, 1.0020) | 0.115 |
| ***P*-value** | 0.8110 |  |  | 0.2852 | 0.1717 | 0.1188 |  |  |
| **High** |  |  |  |  |  |  |  |  |
| **Adjusted β**  **(95% CI)** | -0.0004  (-0.0007, -0.0002) |  | 281 | -0.0005  (-0.0008, -0.0002) | 0.0009  (-0.0006, 0.0024) | 0.0014  (-0.0002, 0.0030) | 0.9971  (0.9720, 1.0222) | 0.088 |
| ***P*-value** | 0.0019 |  |  | 0.0004 | 0.2614 | 0.0906 |  |  |

Note: Adjusted β (95% CI), P-value. Outcome variables: lumber spine BMD. exposure variables: total cholesterol. Adjustment variables: age, sex, race/ethnicity, income to poverty ratio, blood urea nitrogen, total protein, serum uric acid, serum calcium, physical activity and body mass.
